# Cell-free expression tools to study co-translational folding of alpha helical membrane transporters

**DOI:** 10.1038/s41598-020-66097-4

**Published:** 2020-06-04

**Authors:** Nicola J. Harris, Grant A. Pellowe, Paula J. Booth

**Affiliations:** King’s College London, Department of Chemistry, Britannia House, 7 Trinity Street, London, SE1 1DB UK

**Keywords:** Lipids, Protein folding, Membrane biophysics

## Abstract

Most helical membrane proteins fold co-translationally during unidirectional polypeptide elongation by the ribosome. Studies thus far, however, have largely focussed on refolding full-length proteins from artificially induced denatured states that are far removed from the natural co-translational process. Cell-free translation offers opportunities to remedy this deficit in folding studies and has previously been used for membrane proteins. We exploit this cell-free approach to develop tools to probe co-translational folding. We show that two transporters from the ubiquitous Major Facilitator Superfamily can successfully insert into a synthetic bilayer without the need for translocon insertase apparatus that is essential *in vivo*. We also assess the cooperativity of domain insertion, by expressing the individual transporter domains cell-free. Furthermore, we manipulate the cell-free reaction to pause and re-start protein synthesis at specific points in the protein sequence. We find that full-length protein can still be made when stalling after the first N terminal helix has inserted into the bilayer. However, stalling after the first three helices have exited the ribosome cannot be successfully recovered. These three helices cannot insert stably when ribosome-bound during co-translational folding, as they require insertion of downstream helices.

## Introduction

Classic folding studies measure the structure change between the folded state and a partially unfolded state, usually using overexpressed, purified protein^1–5^. While providing important thermodynamic and kinetic information on the final stages of folding, these studies cannot replicate the co-translational process that occurs in the cell, where folding occurs during elongation of the nascent chain by translating ribosomes. *In vivo*, most alpha helical membrane proteins insert and fold co-translationally into the membrane via the Signal Recognition Particle (SRP) and translocon apparatus^6–9^. Co-translational folding can be accessed *in vitro* by expressing the protein of interest using a cell-free system, allowing direct control and measurement of folding whilst the nascent chain is being synthesised.

The study of co-translational insertion and folding of membrane proteins is in its infancy, and very few proteins have been investigated. Most work in the area has addressed insertion of individual transmembrane (TM) helices via the translocon, and the topogenesis of these helices has been investigated via a combination of different methods including proteolysis, glycosylation, cysteine accessibility and photocrosslinking^10–14^. However, until recently there were no investigations into the subsequent structure formation as the protein folds co-translationally in the bilayer. Following an influential study of the co-translational folding of bacteriorhodopsin^15^, we introduced an approach to investigate the co-translational insertion and folding of TM helices in the bilayer, using well-behaved, well-characterised, stable proteins for our initial work (the rhomboid protease GlpG and disulphide bond reducing protein DsbB)^16^. We found that insertion and folding in the lipid bilayer can occur spontaneously and with high efficiency, in the absence of any chaperones or insertase apparatus such as the translocon^16,17^. This, and other work^8,18–20^, indicates that in the absence of a translocon *in vitro*, insertion correlates with the hydrophobicity of individual TM helices. Manipulation of the lipid composition can increase the efficiency of insertion in the absence of a translocon, as demonstrated for GlpG, DsbB^16^, the human endothelin B receptor^21^, the β1-adrenergic receptor^22^, and a number of others^23–28^. These studies set a precedent for extending our cell free, co-translational approach to larger membrane proteins to ascertain whether dynamic, multidomain proteins can also insert spontaneously into lipid bilayers and fold correctly in the absence of translocon apparatus. Here, we successfully advance our approach to representative members of the Major Facilitator Superfamily (MFS) of transporters, providing new tools to probe their co-translational folding. The MFS is one of the largest transporter families, with MFS proteins being ubiquitous across eukaryotes and prokaryotes as they transport many essential nutrients and waste products in and out of cells^29^.

The MFS transporter LacY (lactose permease) has previously been made cell-free, in a cell extract-based system which contained the translocon^30,31^. There have also been a number of single-molecule force spectroscopy studies on LacY, which probed the interactions in the bilayer between individual TM helices both with and without the translocon^32,33^. In this paper we add to previous studies by using PURExpress, a recombinant transcription/translation system devoid of translocon or folding chaperones^34^ to show that the 12 TM MFS transporters LacY and XylE are able to spontaneously insert into liposomes with high efficiency, in the absence of a translocon. The use of cell-free expression enables the lipid dependence of co-translational insertion and folding of these transporters to be probed. It is well established that altering the lipid composition of the bilayer can alter *in vitro* folding^4,35–37^ and function^38–41^ of membrane proteins, although there are also examples of proteins which are not dependent on lipid composition^23,42^. There is extensive knowledge of the effect of the lipid bilayer composition on LacY with 1,2-dioleoyl-sn-glycero-3-phosphoethanolamine (DOPE) being required for correct folding, function and topology^17,37,43–48^. A previous study, which contained the translocon, used cell-free expression with membrane vesicles derived from a PE-lacking *E. coli* strain. This study found that PE is essential for the correct folding of LacY. Addition of PE post-translationally was able to correct the conformation of misfolded LacY^31^.

Here, we investigate the lipid dependence of LacY and XylE cell-free co-translational folding, by first using phosphocholine lipids as a neutral reference bilayer and measuring the insertion efficiency of each transporter in 1,2-dimyristoyl-sn-glycero-3-phosphocholine (DMPC) and 1,2-dioleoyl-sn-glycero-3-phosphocholine (DOPC). *E. coli* inner membranes, where LacY and XylE naturally reside, do not contain PC lipids, but mainly comprise ~70% PE with ~25% negatively charged phosphatidylglycerol (PG) lipids^49,50^. Thus the effect of PG and PE lipids on protein insertion was also investigated. Lipids with PE headgroups form non-lamellar phases in aqueous solutions, where monolayers have a tendency to curve towards the aqueous phase. Constraining PE monolayers in a bilayer by mixing with a bilayer-forming lipid (such as DOPC or 1,2-dioleoyl-sn-glycero-3-phosphoglycerol (DOPG)) causes high outward lateral chain pressure, and a corresponding decrease of lateral pressure in the headgroup region. We found that changing the lipid composition of the liposomes alters the yield of LacY and XylE in the bilayer, with an increase in DOPE and DOPG improving the yield of both transporters.

MFS transporters have two pseudo-symmetrical domains, the N and C domains, and previous work^3,43^ has shown that in LacY the N domain is more stable than the C domain. The two domains of LacY have been expressed *in vivo* as two separate entities^51–53^, and were found to be susceptible to protease digestion unless co-expressed together^51^. In addition, the C domain was less stable when expressed alone^52^. The cell-free approach can be exploited to probe the expression and co-translational folding of individual MFS domains as separate polypeptides *in vitro*. This has been performed previously to express the N domain of LacY alone in a cell extract-based cell-free system, with native membranes which had translocon present^30^. In this paper we probe the independent stability of the two domains of LacY and XylE when expressed as separate polypeptides cell-free using the PURE system in the presence of liposomes without any translocon. We show that each N and C domain of both LacY and XylE can stably integrate into a bilayer with high efficiency when expressed individually, unaided by the translocon.

We have further taken advantage of the cell-free method by directly influencing folding during translation. We have devised an approach to pause and re-start LacY translation after defined numbers of transmembrane helices have been synthesised, and elucidate the effect on the ability to continue folding the full-length protein. Pausing and re-starting translation can be achieved by the omission of a single amino acid from the cell-free expression components, causing translation to halt when it reaches the first codon of the missing amino acid due to the lack of availability of its cognate aminoacyl-tRNA. Tryptophan (Trp) is an ideal candidate to omit from the cell-free reaction, as it has only one codon (TGG). Native Trp residues are usually found at the bilayer interface^54–56^ and can be readily mutated, minimising potential disruption to helix-helix interactions. Trp is not essential for function in LacY^57^. Although W151 does form part of the substrate binding pocket, LacY is functional when W151 is replaced by Phe or Tyr^58^. A Trp-less LacY has previously been studied with all native Trps changed to either Phe or Tyr^3^. Here, we replace Trp with Phe, as the Tyr mutations were found to stabilise LacY and may alter folding pathways. The pause and re-start approach demonstrated here sets the scene for future investigations probing whether helices insert and pack correctly in sequence order, or require later C terminal regions to be translated for correct folding.

## Results

### Cell free synthesised LacY and XylE insert spontaneously into liposomes

**Figure 1:**
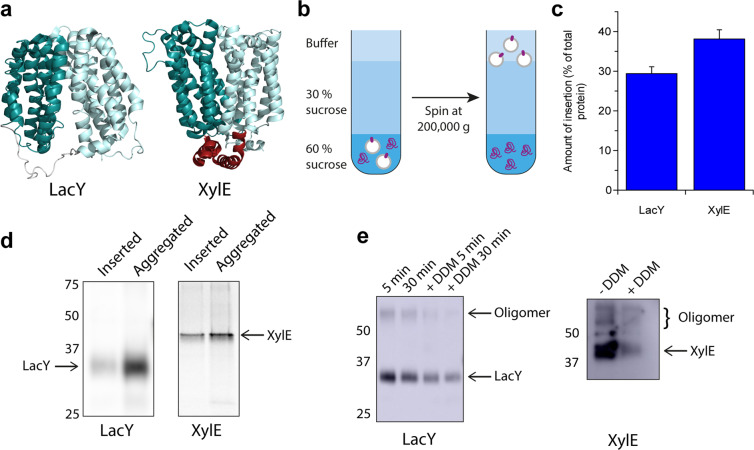
The MFS transporters LacY and XylE spontaneously insert into liposomes when expressed cell-free. (**a**) Crystal structures of LacY and XylE (PDB codes 2V8N^64^ and 4GBY^65^). Both have the same two domains of 6 helices, XylE has an additional cytoplasmic domain important for function (coloured red). (**b**) Schematic of sucrose flotation. The cell-free reaction is mixed with 60% (w/v) sucrose and spun at 200,000 g. The liposomes containing inserted protein float to the buffer:30% sucrose interface, aggregated/misfolded/non-inserted protein and the cell-free kit components remain in the bottom fraction. (**c**) LacY and XylE were expressed cell-free in the presence of 25:50:25 DOPC:DOPE:DOPG liposomes and a sucrose gradient performed as described in (b). The top (inserted) and bottom (aggregated) fractions were quantified via liquid scintillation counting of incorporated [^35^S] methionine, and the efficiency of insertion calculated by expressing the % of protein in the top fraction compared to the total protein synthesised in the cell-free reaction. LacY inserted with a yield of 29% and XylE with a yield of 38%. (**d**) SDS-PAGE of the top (inserted) and bottom (aggregated) fractions of a sucrose gradient following cell-free expression. The gels are from two different experiments; LacY was detected via an anti-HA tag antibody, XylE by phosphorimaging of incorporated [^35^S] methionine. (**e**) LacY and XylE were made via cell-free expression in the presence of 25:50:25 DOPC:DOPE:DOPG liposomes. Following flotation on a sucrose gradient, LacY was incubated at 25 °C with 20 ng of thermolysin in 40 mM HEPES-KOH pH 7.6 for 5 or 30 min, either with or without 2% DDM to solubilise the liposomes. XylE was incubated overnight at 4 °C with 10 ng thermolysin in 40 mM HEPES-KOH pH 7.6, either with or without 2% DDM. Both transporters were more digested when solubilised in DDM, but protected from proteolysis when in liposomes, indicating stable insertion into the bilayer. A small amount of oligomer was observed for each protein, which was also susceptible to proteolysis. Protein was detected via an anti-HA tag (LacY) or anti-His tag (XylE) antibody. Representative gels are shown (see Supplementary Information for an example repeat gel with the same trend). The gels in this figure are cropped from the original images but otherwise unadjusted. The original gels for all of those in this figure are in the Supplementary Information and the related gel intensities are in Fig. S2.

The *E. coli* MFS sugar transporters LacY and XylE (Fig. 1a) were synthesised cell-free using PURExpress. Liposomes composed of a 25:50:25 mol ratio of DOPC:DOPE:DOPG were supplied during synthesis, as we have found previously in studies of purified LacY that this lipid composition supports correct folding and function^17,37^. The liposomes were added to the cell-free reaction prior to initiation by addition of DNA. Following cell-free expression, the liposomes were floated on a sucrose gradient containing 4 M urea. We have shown in earlier work that liposomes and proteoliposomes, containing correctly inserted, folded protein, float to the top of the gradient and are separated from PURExpress components and misfolded, aggregated, non-inserted protein which remain at the bottom of the sucrose gradient^16^ (Fig. 1b). Our prior work on GlpG and DsbB established that all of the protein in the top floated fraction of the sucrose gradient was in one orientation in the liposome and correctly folded, with the same activity as protein expressed, purified and reconstituted from detergent micelles after being extracted from membranes in a folded state^16^. We therefore refer to this top fraction as inserted protein; representing correctly inserted, folded protein. The sucrose gradient was fractionated into a top (inserted) and bottom (aggregated) fraction, and the amount of cell-free synthesised protein in each detected by either western blotting or quantified by liquid scintillation counting (LSC) of incorporated [^35^S]-methionine. Folded LacY runs as a diffuse band on SDS-PAGE when produced cell-free (Fig. 1d), as previously observed and attributed to the high salt content of the reaction^31^. As the bottom fraction is misfolded protein it is likely that it is in a different conformation and runs differently, and less diffuse on SDS-PAGE gels, than the top folded fraction. We therefore quantify the amount of protein in the top and bottom sucrose fractions by LSC. The efficiency of insertion was then expressed as a percentage of the total amount of protein synthesised, in which 100% would mean that all the protein synthesised was inserted and folded correctly into liposomes and present in the top, inserted, fraction of the sucrose gradient. Conversely, 0% would mean that all the protein synthesised was found in the bottom fraction of the sucrose gradient and was aggregated and/or misfolded. Both LacY and XylE were found to insert into 25:50:25 DOPC:DOPE:DOPG liposomes with a yield of 29% and 38%, respectively (Fig. 1c,d). Consistent with their inserted state, the proteins in the top fraction were resistant to urea extraction. We have previously demonstrated that LacY expressed cell-free using PURExpress in this lipid composition of 25:50:25 DOPC:DOPE:DOPG is capable of uphill transport, and is therefore functional and correctly folded^17^. Protection from proteolysis also indicates whether a membrane protein is stably integrated into a bilayer (Fig. S1). LacY and XylE from the top fraction of the sucrose gradient were incubated with thermolysin. Both proteins were found to be resistant to protease digestion, as would be the case for purified protein reconstituted into liposomes (Fig. S1). When solubilised in n-Dodecyl-β-D-Maltopyranoside (DDM) LacY and XylE were susceptible to proteolysis, indicating exposure of protease sites (Fig. 1e). This protease protection in liposomes indicates stable integration of cell-free produced LacY and XylE into the bilayer.

### Lipid dependence of cell-free spontaneous insertion

**Figure 2:**
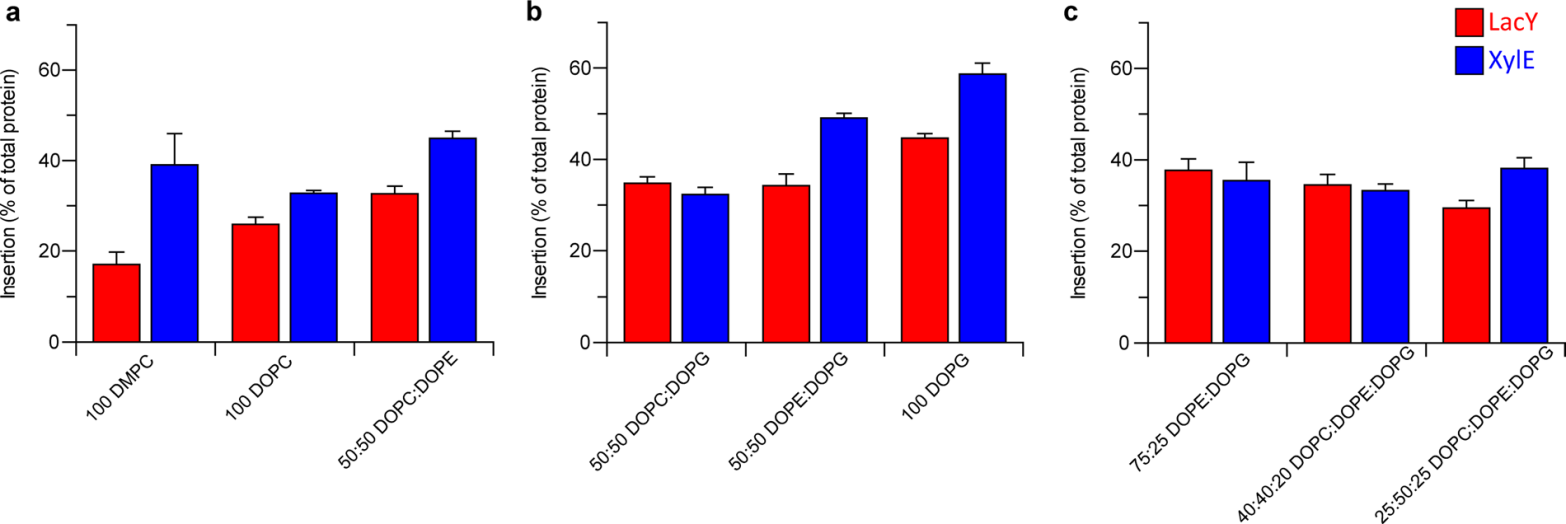
Altering the lipid composition can increase the yield of spontaneous insertion. LacY and XylE were expressed cell-free in the presence of liposomes of different lipid compositions. The inserted protein was quantified via liquid scintillation counting of incorporated [^35^S] methionine, and the efficiency of insertion calculated by expressing the % of protein in the top fraction of the sucrose gradient compared to the total protein synthesised in the cell-free reaction. (**a**) The lateral chain pressure of the lipids was increased by addition of DOPE. (**b**) Charge was increased in the headgroup region by addition of DOPG. (**c**) Insertion into 75:25 DOPE:DOPG (similar to the native *E. coli* inner membrane), 40:40:20 DOPC:DOPE:DOPG and 25:50:25 DOPC:DOPE:DOPG. Errors are SEM, data is the result of a minimum of 2 repeats (50:50 PE:PG, n = 5).

The lipid dependence of LacY and XylE cell-free co-translational folding was assessed. First, insertion was measured in a DMPC bilayer. Lateral pressure was then increased by switching the bilayer composition from all DMPC to all DOPC, then increased further by adding DOPE to DOPC at a 50:50 mol ratio. Then, to determine the effect of charged headgroups on insertion yield, DOPG was added to DOPC bilayers to give a 50:50 ratio of DOPC:DOPG. The combination of DOPE and DOPG together was also measured (50:50 DOPE:DOPG), as well as a bilayer comprised solely of charged headgroups (pure DOPG). Finally, the insertion yield into a composition more similar to the *E. coli* inner membrane (75:25 DOPE:DOPG) was measured, and compared to two ternary mixtures containing a majority of PG and PE over PC; 25:50:25 DOPC:DOPE:DOPG (as shown in Fig. 1) and 40:40:20 DOPC:DOPE:DOPG. The insertion yield of LacY and XylE was determined from the amount of protein in proteoliposomes that floated to the top of the sucrose gradient, and is given as a percentage of the total protein synthesised (Fig. 2 and Table 1).

**Table 1:** Amount of inserted LacY and XylE in each lipid composition.

Lipid composition	Amount of inserted protein (% of total protein expressed)	
	LacY	XylE
100 DMPC	17.0 ± 2.7	39.0 ± 7.0
100 DOPC	25.9 ± 1.6	32.8 ± 0.6
50:50 DOPC:DOPE	32.7 ± 1.7	44.9 ± 1.6
50:50 DOPC:DOPG	34.8 ± 1.4	32.3 ± 1.5
50:50 DOPE:DOPG	34.3 ± 2.5	49.1 ± 1.0
100 DOPG	44.7 ± 1.0	58.7 ± 2.4
75:25 DOPE:DOPG	37.7 ± 2.5	35.5 ± 4.0
40:40:20 DOPC:DOPE:DOPG	34.5 ± 2.4	33.3 ± 1.4
25:50:25 DOPC:DOPE:DOPG	29.4 ± 1.7	38.1 ± 2.3

The yield of inserted LacY and XylE is dependent on the lipid composition of the supplied liposomes. Errors are SEM, data is the result of a minimum of 2 repeats (50:50 PE:PG, n = 5).

The LacY insertion yield was found to increase when a pure DMPC bilayer was changed to pure DOPC, from 17.0 ± 2.7% to 25.9 ± 1.6%. The incorporation of DOPE into the bilayer to give a 50:50 DOPC:DOPE composition produced a further but modest increase in insertion yield over DOPC alone, at 32.7 ± 1.7%. An increase in lateral pressure therefore increased the insertion yield of LacY.

To determine the effect of a charged headgroup on the insertion yield of LacY, DOPG was added to DOPC bilayers to give a 50:50 ratio of DOPC:DOPG, which had a yield of 34.8 ± 1.4%. The insertion yield in 50:50 DOPE:DOPG was measured, combining charged headgroups with an increased lateral chain pressure. The insertion yield in 50:50 DOPE:DOPG was 34.3 ± 2.5%, comparable to that in 50:50 DOPC:DOPG (Fig. 2). In a pure DOPG bilayer, the insertion yield increased to 44.7 ± 1.0%.

A composition more similar to the *E. coli* inner membrane, 75:25 DOPE:DOPG, was also measured, as well as two ternary mixtures containing a majority of PG and PE over PC; 25:50:25 DOPC:DOPE:DOPG (as shown in Fig. 1) and 40:40:20 DOPC:DOPE:DOPG. 75:25 DOPG:DOPE was found to be best (37.7 ± 2.5%, Fig. 2, Table 1).

The same lipid compositions were used to investigate XylE insertion. Conversely to LacY, the insertion yield of XylE did not change when lateral pressure was increased. No significant increase in insertion occurred when DMPC was switched to DOPC (39.0 ± 7.0% compared to 32.8 ± 0.6%, so within the SEM of the DMPC measurement, Fig. 2 and Table 1), or when DOPE was incorporated into the bilayer to give a 50:50 DOPC:DOPE composition (44.9 ± 1.6% in 50:50 DOPC:DOPE, again within the SEM of the DMPC measurement).

To determine the effect of charged headgroups on XylE insertion efficiency, 50:50 DOPC:DOPG and 50:50 DOPE:DOPG were measured. The insertion efficiency was 32.3 ± 1.5% in 50:50 DOPC:DOPG and 49.1 ± 1.0% in 50:50 DOPE:DOPG. Combining charged headgroups and lateral pressure therefore produces a small increase in insertion efficiency compared to DMPC (39.0 ± 7.0%). A pure DOPG bilayer produced the largest increase in insertion efficiency at 58.7 ± 2.4% (Fig. 2), and none of the other compositions tested were an improvement on this yield (Table 1). The highest insertion yield for both LacY and XylE was therefore produced in pure DOPG bilayers.

### Cell free expression of stable, individual transporter domains

**Figure 3:**
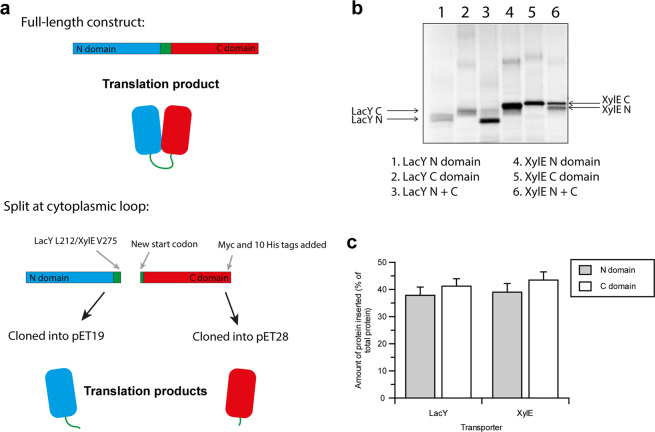
Cell-free expression of individual MFS domains as two separate polypeptides. (**a**) The DNA for each domain of LacY and XylE were cloned into two separate plasmids to be expressed as independent domains. LacY was split at residue L212, XylE at V275. The N and C domain of each MFS transporter could then be expressed separately as two separate polypeptides in PURExpress. (**b**) The N and C domains of LacY and XylE were expressed individually using PURExpress in the presence of 25:50:25 DOPC:DOPE:DOPG liposomes, and the inserted protein was separated from aggregates by sucrose flotation. When the inserted protein was analysed by SDS-PAGE and visualised by [^35^S] Met phosphorimaging, a band was observed for the N and C domains of each transporter, indicating that each can insert spontaneously into liposomes when produced separately. A small amount of oligomer was also observed for the LacY C domain and the N and C domains of XylE. The N and C domains of each transporter were also expressed as two separate polypeptides in the same reaction and floated on a sucrose gradient (lanes 3 and 6). The original, uncropped, image is in the Supplementary Information, and is otherwise unadjusted. (**c**) As in (b), but the amount of spontaneous insertion was quantified via LSC of incorporated [^35^S] Met. Around 40% of the protein expressed inserts spontaneously into liposomes under these conditions (LacY N 37.9 ± 3.0%, LacY C 41.2 ± 2.8%, XylE N 39.0 ± 3.2%, XylE C 43.4 ± 3.0%. Error is SEM, n = 3).

MFS transporters are composed of two pseudo-symmetrical domains. Whether or not these domains are able to express and insert independently was investigated by splitting each transporter in half, so the transporter could be expressed as two separate polypeptides comprising the two separate domains (Fig. 3a). These we called the ‘N domain’ (from residue 1, including TMs 1-6 and most of the hydrophilic connecting loop), and ‘C domain’ (the remainder of the protein; the rest of the connecting loop, and TMs 7-12). The LacY N domain was comprised of residues 1-212, and XylE of residues 1-275. The two domains of each transporter were then cloned into two separate plasmids to be expressed individually.

Previous work has shown that when the two domains of LacY were expressed *in vivo* as two separate entities^51–53^, they were unstable and susceptible to protease digestion unless co-expressed together^51^. We found that *in vivo* expression of the two domains of XylE yielded similar results, with a very low yield of protein and the presence of protein aggregates when the domains were expressed independently (Fig. S3).

**Figure 4:**
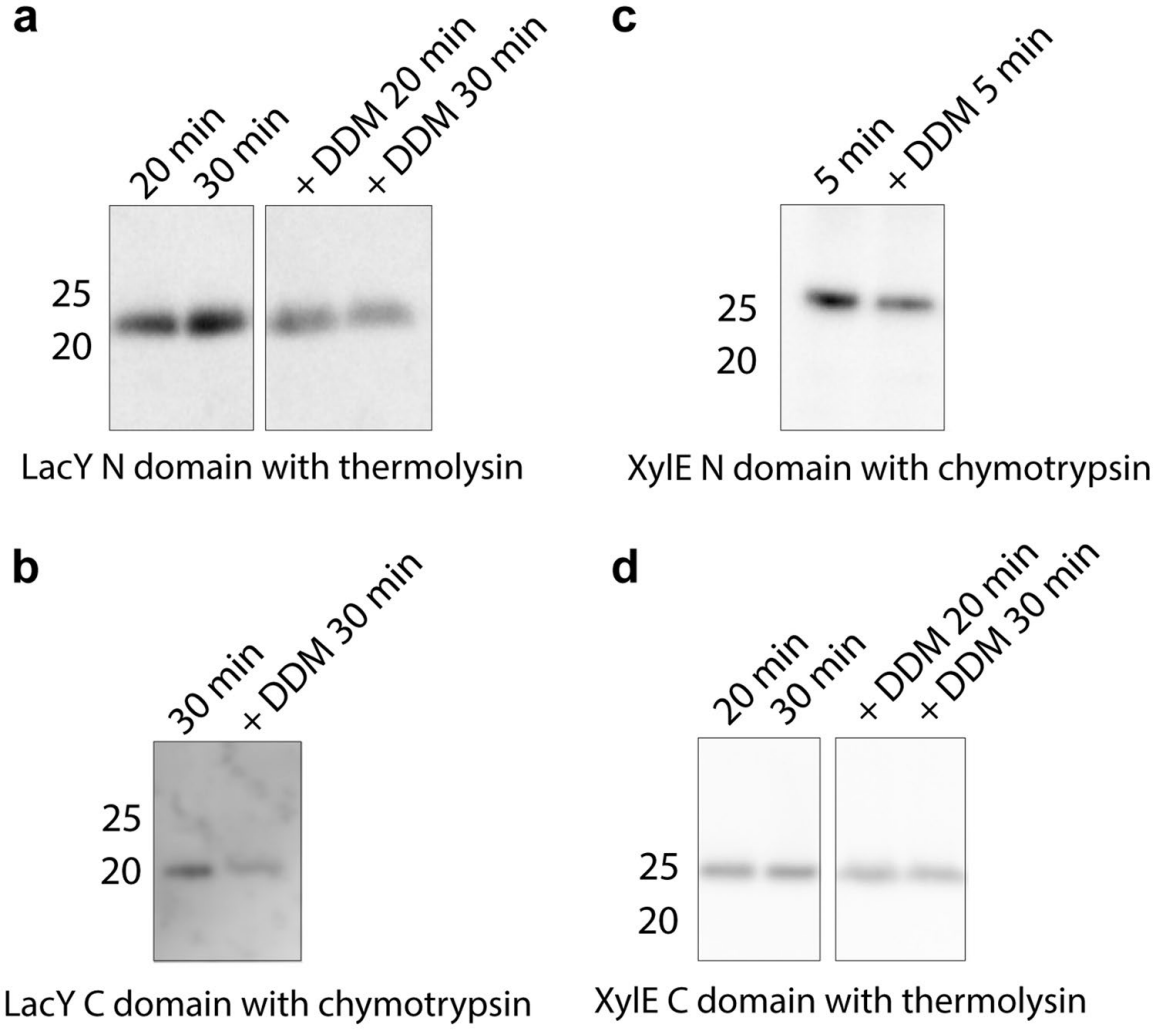
Proteolysis resistance of individual MFS domains. The N and C domains of LacY and XylE were made via cell-free expression in the presence of 25:50:25 DOPC:DOPE:DOPG liposomes. Following flotation on a sucrose gradient, each individual domain was incubated at 25 °C with either 20 ng of thermolysin or chymotrypsin in 40 mM HEPES-KOH pH 7.6 for the time points indicated on the gels, both with and without 2% DDM. (**a**) LacY N domain incubated with thermolysin for 20 or 30 min, (**b**) LacY C domain incubated with chymotrypsin for 30 min, (**c**) XylE N domain incubated with chymotrypsin for 5 min, (**d**) XylE C domain incubated with thermolysin for 20 or 30 min. Each domain was more digested when solubilised in DDM, but was protected from proteolysis when in liposomes, indicating stable insertion into the bilayer. Protein was detected via an anti-His tag antibody. Selected time points are shown here, the original uncropped gels and relative band intensities for all lanes are in Fig. S4. Representative gels are shown (see Supplementary Information for example repeat gels with the same trend).

The N and C domains of LacY and XylE were each expressed either in separate cell-free reactions using PURExpress in the presence of 25:50:25 DOPC:DOPE:DOPG liposomes and floated on a sucrose gradient to obtain inserted domains, or the N and C domains of each protein were expressed together in the same reaction. The separate N and C domains of LacY and XylE floated to the top fraction of the sucrose gradient, indicating spontaneous insertion of the individual domains into liposomes in all cases (Fig. 3b,c). When analysed by SDS-PAGE, each domain was predominantly monomeric with some oligomeric species observed, and ran below its expected molecular weight, an indication that it has some folded structure^59^. Each individual domain was also resistant to urea extraction and proteolysis. To measure the degree of protease protection, each inserted domain from the top fraction of the sucrose gradient was incubated with either thermolysin or chymotrypsin. Each domain was found to be resistant to protease digestion, whereas the top fraction solubilised in DDM was susceptible to proteolysis (Fig. 4, band intensities are in Fig. S4). This, as was the case for the full-length transporters, indicates that the individual transporter domains stably integrate into the bilayer when produced by cell-free expression. The yield of insertion of the independent domains was similar to the full-length transporters in the same lipid composition (~35% full length compared to ~40% independent domains for both transporters).

Cell-free expression is therefore a viable method for the co-translational study of individual domains of transporters, as they are produced in good yields (1-3 µg per 25 µl PURExpress reaction synthesised, the same as full-length transporters) and spontaneously insert into liposomes.

### Pausing and re-starting translation of LacY

A further advantage of cell-free expression is the ability to pause translation. We sought to demonstrate this approach with LacY, as it is a very well characterised protein for which there is a large amount of information on its stability and folding, and is known to tolerate Trp mutations^3,57^. A Trp-free version of LacY with an N-terminal HA tag was constructed, and 2 sites in the N domain were chosen to pause translation. The sites chosen (Fig. S5) are predicted to produce a nascent chain with either 1 TM helix (called NC(TM1)) or 3 TM helices (called NC(TM3)) outside the ribosome while remaining ribosome attached (~60 residues fit inside the ribosome exit tunnel when in an alpha helical conformation^60^). These single-Trp sites were I103W for the NC(TM1) construct, located in the periplasmic loop between TM3 and TM4, and the native Trp residue W171 for the NC(TM3) construct, located towards the periplasmic end of TM6. Omitting Trp from the cell-free reaction for these constructs should therefore produce LacY for which 1 (I103) and 3 (W171) TM helices have exited the ribosome, and thus can insert and fold in the liposome.

The constructs NC(TM1) and NC(TM3) were synthesised in PURExpress without the amino acid Trp (using the Δaa, tRNA kit), and inserted protein separated by flotation as previously on a sucrose gradient with 4 M urea. As these constructs lack a stop codon, the expressed protein remains ribosome attached. Versions of NC(TM1) and NC(TM3) were also made with a stop codon in place of the Trp codon, to allow release from the ribosome.

**Figure 5:**
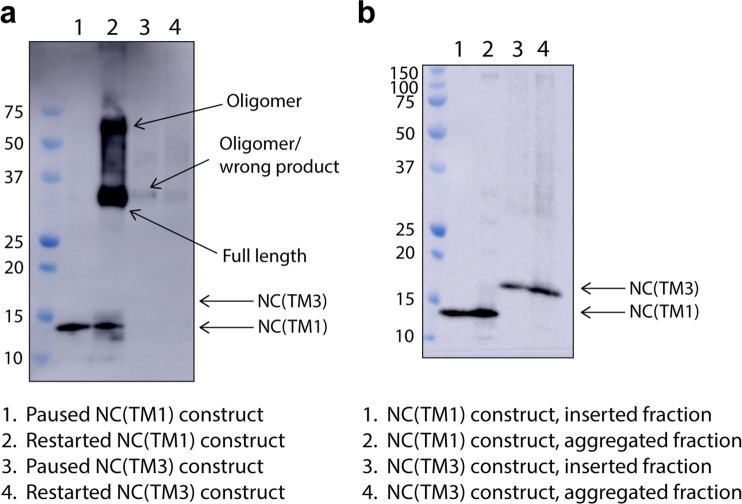
LacY translation paused and restarted at different positions along the polypeptide. (**a**) LacY NC(TM1) and NC(TM3) translation. Lanes 1 and 3 show the NC(TM1) and NC(TM3) constructs when Trp was omitted from the cell-free reaction, stopping translation. Lanes 2 and 4 show the NC(TM1) and NC(TM3) constructs when Trp was added back into the cell-free reaction after a 30 min incubation. Following expression, each was floated on a sucrose gradient containing 4 M urea to establish whether the protein made was liposome associated. The floated, i.e. inserted, fractions were analysed by SDS-PAGE. Oligomeric species can be observed in lanes 2, 3 and 4, indicating a competing aggregation pathway. LacY which has stopped translation at NC(TM1) (lane 1) inserts into the liposome and is not removed by urea. When Trp is added back into the cell-free reaction (lane 2), translation continues, but a truncated protein band remains. No truncated bands are observed when translation is stopped at NC(TM3) (lane 3), an indication that LacY of this length is not stably associated with the liposome when still attached to the ribosome. A low intensity band is observed (slightly larger than full-length LacY), an indication of aggregation or the result an incorrect translation product. Larger aggregates are unlikely to enter the SDS-PAGE gel. Consistent with this observation, very little full-length inserted protein is observed when translation is continued (lane 4), presumably because the initial polypeptide was not stably associated with the liposomes, and due to further aggregation. (**b**) The same constructs were expressed, but with stop codon instead of a Trp residue i.e. released from the ribosome. The top (inserted) and bottom (aggregated) fractions were analysed by SDS-PAGE. The NC(TM1) construct runs at its expected MW of 14 kDa, the NC(TM3) construct runs at ~16 kDa, well below its expected MW of 21 kDa. Both insert spontaneously into liposomes, NC(TM1) better than NC(TM3) as it has higher band density. Each polypeptide was detected by western blot via an N-terminal anti-HA tag antibody. All of the lanes shown in this figure are uncropped.

A band for the NC(TM1) construct was observed in the top fraction of the sucrose gradient when analysed by SDS-PAGE (Fig. 5). This indicates that the NC(TM1) construct floated with the liposome on a sucrose gradient, and that TM1 is able to insert into the liposome when bound to the ribosome. The presence of the NC(TM1) construct in the top floated fraction shows that NC(TM1) inserted whether the nascent chain was still attached to the ribosome or not (i.e. independent of whether there is a Trp or a stop codon, see Fig. 5a lane 1 compared to Fig. 5b lane 1). With the ribosome bound, TM2 and 3 of the NC(TM1) construct are within the ribosome tunnel, but with a stop codon present helices TM2 and 3 are released from the ribosome. Thus, the results indicate that TM2 and TM3 are able to follow TM1 into the bilayer once released from the ribosome. In contrast, the NC(TM3) construct did not insert into the liposome, as only a faint oligomer (or an incorrect translation product, indicated in Fig. 5a lane 3) band was observed. However, a band for the NC(TM3) construct was observed upon release of the ribosome with a stop codon (Fig. 5b lane 3). Thus, the first 3 TM helices do not insert into the bilayer as a nascent chain attached to the ribosome. However, after release from the ribosome these helices can insert, suggesting that during co-translational folding, TM 2 and 3 require downstream helices to be synthesised in order to insert into the bilayer.

Stopping translation by omitting Trp from the cell-free reaction means that translation can be restarted by adding excess Trp once translation has stalled. Trp was added to cell-free reactions of the NC(TM1) and NC(TM3) constructs, which had initially been incubated for 30 min in the absence of Trp. The reaction was continued for 8 min after Trp was added (full length LacY is produced in 10 min, Fig. S6), followed by incubation on ice and mixing with 60% sucrose and 4 M urea to halt translation.

Translation of LacY could be restarted when paused in the NC(TM1) construct, as shown by a band for the full-length protein (see Fig. 5a, lane 2). Although bands were always observed for the stalled and restarted NC(TM1) construct, the intensities of the bands varied between experiments (for example see Fig. S7) as the gels where ribosomes were present do not always transfer well during blotting. Oligomers were also often seen in the restarted sample (see Fig. 5a, lane 2). Such a large proportion of oligomeric species is not usually observed for cell-free produced protein (see Fig. 1), so in this instance indicates a higher propensity for aggregation when translation has been paused. The re-initiation of translation is not completely efficient as a truncated band remains on the SDS-PAGE gel together with that of the full-length protein (Fig. 5). The continuing presence of the paused construct (Fig. 5a, lane 2) is likely attributed to translation failing to re-start once Trp is added.

When translation was paused in the NC(TM3) construct and Trp added to enable translation to resume no new bands were observed, indicating that re-starting translation from this position does not produce full-length protein in the bilayer (Fig. 5a, lane 4). This lack of full-length protein is a good indication that the full-length band observed for the NC(TM1) construct (Fig. 5a, lane 2) is not simply protein which has translated from the start at the N terminus, else it would be observed for the NC(TM3) construct also. Quantification of the total amount of NC(TM1) and NC(TM3) produced showed a similar amount of each was made (Fig. S8), indicating a large amount of aggregation of the NC(TM3) construct.

## Discussion

We have shown that cell-free expression is a highly useful and adaptable tool for investigations of the co-translational folding of membrane transporters. The MFS transporters LacY and XylE can be synthesised cell-free in the absence of chaperones and translocon and insert spontaneously into the liposomes supplied during expression. This insertion is efficient, with up to ~58% of the MFS protein synthesised being inserted into the bilayer under the lipid compositions studied. These high yields compare favourably to those of ~47% for the smaller, single domain protein DsbB and ~68% for GlpG^16^. Cell-free expressed LacY and XylE are both monomeric on SDS-PAGE, resistant to urea extraction and resistant to protease degradation (Fig. 1), a good indication that that they are stably integrated into the bilayer. Our previous work^17^ has shown that LacY expressed cell-free in the same manner as reported here, in 25:50:25 DOPC:DOPE:DOPG lipids, is functional and able to transport substrate uphill against a concentration gradient. We have also previously shown that cell free expression results in correctly folded, functional proteins (e.g. GlpG and DsbB) in one orientation in liposomes^16^. Moreover, both these latter proteins inserted and folded co-translationally in the bilayer.

Cell-free insertion of LacY and XylE exhibits lipid composition dependencies. An increase in lateral chain pressure (i.e. increasing DOPE) and addition of charged headgroups was found to be beneficial for LacY insertion. The largest increase in the XylE insertion yield occurred in a negatively charged bilayer (i.e. DOPG). These results together indicate that a favourable interaction with the headgroup region of the bilayer promotes insertion. This effect appears to be non-specific, as either the addition of charged headgroups (DOPG) or a decrease of headgroup lateral pressure (DOPE) both promote insertion of LacY and XylE. Previous work found that GlpG also prefers lipids with charged headgroups and high lateral chain pressure. Conversely the highest insertion efficiency for DsbB was observed in a pure DMPC bilayer, which has low lateral pressure and neutral headgroups^16^. A similar effect has been observed for bacteriorhodopsin, in which an increase in lateral pressure caused by the addition of DOPE to DOPC bilayers inhibited insertion. In this case, longer acyl chains were found to inhibit insertion, and charged headgroups had no effect^27^. Although pure DOPG bilayers had the highest insertion efficiency for both LacY and XylE, highly charged bilayers are unable to hold an ion gradient which makes them unsuitable for many downstream assays^17^. A lack of DOPE also causes LacY to form the wrong topology, causing the N domain to be inverted with respect to the C domain^37,45^. The yield of insertion is never higher than ~ 60% efficient for both transporters, even in an *E. coli*-like bilayer (75 DOPE: 25 DOPG). A larger increase in insertion in PE would perhaps be expected given its essential role in folding and function, particularly for LacY^37^. It may be that the translocon SecYEG is critical for insertion and folding in the *E. coli* membrane.

Expression of individual domains *in vivo* can frequently lead to their degradation as their low stability makes them a target for cellular quality control mechanisms^61^. A cell-free approach can be used to express domains individually as has previously shown for the N domain of LacY. Previous work using a cell-extract based cell-free system^30^ investigated N domain stability when ribosome-attached or ribosome-released, and found the N domain was not stable when ribosome-attached, but was stable when released from the ribosome. Our PURExpress cell-free approach can be used to express individual domains without the presence of quality control machinery and proteases. We demonstrate this potential by expressing the individual N and C domains of LacY and XylE cell-free; the domains being unstable when expressed separately *in vivo*^51–53^ (Fig. S3). The cell-free expressed domains were resistant to urea extraction and protease degradation (Figs. 3, 4 and Fig. S4), indicating stable integration into the bilayer. As with full-length protein, the domains inserted spontaneously into the bilayer, demonstrating that the translocon is not required *in vitro*. Previous work^3,43^ has shown that the N domain of LacY is more stable than the C domain, but when expressed cell-free in our system they insert into a bilayer with equal efficiency (Fig. 3c). Being able to express domains independently has also demonstrated that the two domains of LacY and XylE do not require their partner domain in order to insert into a bilayer. This was not the case *in vivo*, where the two domains of LacY had to be expressed together^30,51^, and more C domain was observed when an increasing number of N domain helices were co-expressed^52^. Successfully expressing the two domains of LacY cell-free has therefore supported the earlier hypothesis^51^ that the instability of the N and C domains *in vivo* is likely due to their being targeted by cellular quality control mechanisms, rather than a required cooperativity in domain folding. The cell-free expression of individual domains therefore paves the way for the further study of folding cooperativity between transporter domains, which cannot be accessed by expression *in vivo*.

A cell-free approach introduces a method to pause and restart translation at specific points in the protein sequence, through the omission of an amino acid at the start of the reaction and site-specific positioning of the amino acid codon. For LacY, this method demonstrates that when ribosome-bound, the first helix (the ribosome-bound NC(TM1) construct) inserts into liposomes efficiently on its own, but the first 3 helices (the ribosome-bound NC(TM3) construct) do not. These results indicate that when ribosome-bound during co-translational folding, TM 2 and TM 3 seem to require downstream helices to insert into the bilayer. Consistent with this, previous work which expressed truncated LacY cell-free found that the N domain was not stably integrated into the membrane when ribosome-attached. When ribosome-released however, or when subsequent helices were translated, the N domain was more stable^30^. We therefore find that insertion into a bilayer can begin as soon as a single helix has emerged from the ribosome, and translation of the whole protein can occur successfully following a pause in translation once the first helix is inserted into the membrane. However, pausing translation after the first 3 helices have been synthesised does not produce stable protein, suggesting that there are competing aggregation pathways. The translocon may be a means by which such competing aggregation is avoided *in vivo*.

We have demonstrated a number of tools to aid the investigation of MFS transporter folding during synthesis by the ribosome. These include a highly effective method to optimise the yield of insertion simply by altering the lipid composition of the liposomes supplied during cell-free expression. Our work also shows that large multidomain membrane proteins do not require the translocon to insert into lipid membranes *in vitro*, reinforcing similar findings for other membrane proteins^15,16,21–23,62,63^. Thus the essential role of the translocon *in vivo* is not purely for insertion, but is likely connected to the crowded cellular environment, as well as insertion or translocation of charged amino acids. The translocon seems to additionally have a role in topological control, as demonstrated previously for the proteins YidC and LepB. When these proteins were expressed cell-free using the PURE system, they inserted into PC membranes in the opposite topology to *in vivo* unless SecYEG and SecA/B were present during translation^63^.

We have also demonstrated a method to independently express individual transporter domains, and shown that domains which do not express stably *in vivo* can spontaneously insert into liposomes in the absence of a translocon when expressed cell-free. This approach offers the opportunity to probe the cooperativity of domain insertion and folding. As a starting point, our results reveal that the C domains of LacY and XylE have the inherent ability to insert into bilayers independently of the N domain, but this ability is not manifested *in vivo*. A hallmark of the co-translational process is that folding occurs whilst the polypeptide chain is being lengthened. Taking the simplest model, helices are assumed to insert into the membrane in sequence order as they are being made. However there is little evidence for this, nor is it known which N terminal regions of proteins can insert and fold without requiring C terminal sections that have yet to be made. We have devised an approach to stall and re-start co-translational folding at precise points in the protein chain, and coupled this with truncated chain comparisons, to begin to address the requirement of downstream sequences on folding. This method can be applied to other proteins, provided that there is a suitable residue which can be removed from the protein without perturbing structure or function. For this, Trp provides a good candidate as it can often be replaced with Tyr or Phe without detrimental effects on the folding or function of the target protein. Cysteine is also a good candidate for mutagenesis, as it can often be successfully removed and introduced into proteins for site-specific labelling. To apply this method to other proteins, mutation of as few residues as possible with minimal disruption to charged or polar residues is advisable.

The folding field has been heavily dominated by accessible proteins and methods, with helical membrane proteins being avoided due to inherent difficulties in their study largely stemming from their propensity to aggregate and a lack of experimental approaches. Moreover, most existing studies use artificial folding conditions that cannot replicate the unidirectional N- to C-terminus folding that occurs co-translationally in the cell. Recent studies have gained insight into unidirectional N- to C-terminus folding by single-molecule force spectroscopy^32,33^. Co-translational studies of membrane proteins provide an advantage since they avoid contrived means to reversibly unfold a membrane protein whilst avoiding aggregation; a co-translational approach utilises the fact that the protein chain can insert into the membrane as soon as it is made, avoiding the inevitable aggregation of the whole protein outside the bilayer.

## Materials and methods

### Materials

All standard reagents were purchased from Sigma. The PURExpress® *In Vitro* Protein Synthesis Kit, PURExpress® Δaa, tRNA Kit and all molecular biology reagents were purchased from New England Biolabs. Lipids were purchased from Avanti Polar Lipids, and pre-cast SDS-PAGE gels and detergents were purchased from Generon. Other exceptions are included in the text where relevant.

pET28a was used as the vector for the expression of the full-length *E. coli* wild type MFS transporters. This was modified with an N-terminal HA tag or a C-terminal 10-His tag.

### Single Trp LacY mutants

The native Trp residues in WT LacY (in pET28a modified with a C-terminal 10-His tag) were removed and replaced with Phe. Phe rather than Tyr was used to replace native Trp residues, as using Tyr may alter the folding of LacY and Tyr mutants are significantly more stable than WT LacY^3^. A Trp-free version of LacY was used to create the I103W using the Q5 site-directed mutagenesis kit (NEB). While Ile164 is a suitable candidate for mutation to Trp, mutagenesis was inefficient, possibly due to the low GC content in this region. The native residue W171 (~7 residues downstream) was therefore chosen as a pausing site. A version of each construct was also made with a stop codon (codon TAG) replacing the Trp residue. All of the constructs were modified with an N-terminal HA tag with a GSSG linker between the tag and start codon to enable detection of truncated protein.

### Splitting transporters into independent domains

LacY was split at residue L212 (N domain M1-L212, C domain A213-A417 as performed previously), XylE at residue V275 (N domain M1-V275, C domain G276-L491). The ICH domain of XylE was included in the N domain. A new start codon was inserted at the beginning of each C domain. pET19b with an N-terminal His-tag was used as the vector for all N domain constructs, and pET28a modified with a C-terminal myc tag followed by a 10-His tag was used for all C domain constructs.

### Liposome preparation

DOPC, DOPE, and DOPG were dissolved in cyclohexane at 50 mg.ml^−1^ and mixed at the desired ratios. DMPC was dissolved in chloroform at 50 mg.ml^−1^. Liposomes were prepared as described previously^16^.

### Cell-free expression of MFS transporters

Full length LacY and XylE and the N and C domains of each were expressed by PURExpress as described previously^16^. Liposomes were added to the cell-free reaction prior to initiation by addition of plasmid DNA. Following cell-free expression, the cell-free reaction was mixed with 60% (w/v) sucrose, and 30% (w/v) sucrose was layered on top followed by buffer, and the sample spun at 200,000 g. 40 mM HEPES-KOH and 4 M urea were also included in each sucrose gradient layer. The liposomes float to the buffer:30% sucrose interface, aggregated/misfolded/non-inserted protein and the cell-free kit components remain in the bottom fraction.

Quantification of insertion was performed as previously described^16^. Briefly, for detection of protein by western blot, the sucrose gradient fractions were loaded onto a 12% SDS-PAGE gel and transferred onto PVDF membrane. An anti-HA tag (ThermoFisher) or anti-His tag antibody (Sigma) was used to detect cell-free expressed protein. As the top and bottom fractions of LacY may be in different conformations and therefore migrate differently on SDS-PAGE – notably, folded LacY is observed as a diffuse band in cell-free reactions^31^ (see Figs. 1d and S9), quantification of insertion was done by liquid scintillation counting (LSC) of incorporated [^35^S] Methionine. For reactions containing [^35^S] Methionine, 5 µl of each sucrose layer and 1 µl of the total reaction was pipetted onto 0.45 μm MF-Membranes. The amount of protein in each sucrose layer was then quantified by LSC as described previously^16^.

### Stopping and restarting translation of LacY

For stopping and restarting translation of LacY, the single Trp mutant I103W was constructed using the Q5 site directed mutagenesis kit (New England Biolabs) into Trp-free LacY^3^. To make single Trp W171, the other 5 native Trps were removed^3^. These single Trps were then megaprepped (Qiagen) to make plasmid DNA at 1 mg.ml^−1^.

To stop and start translation, the PURExpress Δaa, tRNA Kit was used according to the manufacturer’s instructions, with the amino acid mix substituted for a 19 amino acid (19AA) mix minus Trp. The concentration of each amino acid was kept the same as those supplied in PURExpress, i.e. 0.3 mM final concentration of each. A separate 7.5 mM Trp stock was made to be added separately as appropriate. The cell-free reaction was started by addition of 1 µg of plasmid DNA and incubated at 30 °C for 30 min. 0.3 mM of Trp was then added to the ‘restarted’ reactions and incubation was continued for a further 8 min (Fig. S6) and then incubated on ice to stop translation. The reactions were floated on a sucrose gradient as described previously^16^, with the following modifications. The liposomes were mixed with 80 µl 60% (w/v) sucrose, and 50 µl of 30% (w/v) sucrose was layered on top, followed by 50 µl of 15% (w/v) sucrose and 50 µl of buffer (4 M urea, 40 mM HEPES-KOH pH 7.6 and 10 mM Mg(OAc)_2_ was in all sucrose layers). The sucrose gradients were then spun at 200,000 g to float the liposomes to the 15% sucrose: buffer interface. The sucrose layers were then pipetted off carefully, either to be loaded directly onto an SDS-PAGE gel, or to be used for further analysis. Protein was detected by western blot using an anti-HA antibody, or by quantification by LSC.

### Protease digestion of cell-free produced protein

All protease digestion was performed on protein from the top fraction of a sucrose gradient. Full-length LacY, LacY N domain and XylE C domain were incubated with 20 ng thermolysin in 40 mM HEPES-KOH pH 7.6 for time points ranging from 5 to 30 min at 25 °C. Full-length XylE was incubated at 4 °C overnight with 10 ng thermolysin. LacY C domain and XylE N domain were not susceptible to thermolysin digestion, so were instead digested in 20 ng chymotrypsin for 5 or 30 min in 40 mM HEPES-KOH pH 7.6 at 25 °C. In all cases and for every time point an identical sample was incubated with 2% DDM to solubilise the liposomes prior to protease incubation. Digestion was stopped by flash freezing in liquid N_2_, and the sample was mixed with SDS loading buffer and loaded onto an SDS-PAGE gel. Bands were visualised by western blotting using an anti-His or anti-HA tag antibody. Each protease experiment was repeated at least twice, and the repeats showed the same trend. Example repeat gels are in the Supplementary Information (Figs. S13–S15).

### Expression of individual domains *in vivo*

The vectors containing the LacY or XylE halves were transformed into BL21-AI cells. Cells were cultured in 6 L baffled flasks with 30 μg.ml^−1^ kanamycin until cell density reached an OD_600_ of 0.8. Cells were then induced with 1 mM isopropyl β-D-1-thiogalactopyranoside (IPTG) and 0.1% L-arabinose until growth arrest, harvested and washed in PBS before resuspension in PBS with 10 mM β-mercaptoethanol (βME), 5 mM EDTA, 0.5 mM phenylmethylsulfonyl (PMSF) and cOmplete protease inhibitor tablet and freezing at −20 °C. The cells were defrosted and incubated at room temperature with Benzonase nuclease for 10 min before resting on ice. Thawed cells were then passed through a cell disruptor (Constant Systems) at 25 kPsi, 4 °C. Cell membranes were harvested at 100,000 × g, 4 °C for 30 min. The membrane pellets were solubilised for 2 hr with mixing at 4 °C in solubilisation buffer (50 mM NaPi pH 7.4, 200 mM NaCl, 20 mM imidazole, 10 mM βME, 10% v/v glycerol, 2% DDM one cOmplete EDTA free protease inhibitor tablet and 0.5 mM PMSF). DDM insoluble material was removed by 30 min 100,000 × g. The supernatant was loaded onto a pre-equilibrated 1 ml Ni-NTA column (50 mM NaPi pH 7.4, 2 mM βME, 10% v/v glycerol, 0.05% DDM, 0.1 mM PMSF, 20 mM imidazole). Bound protein was washed with 75 mM imidazole, then protein was eluted in 500 mM imidazole. The 1 ml elution was injected directly onto a Superdex 10/30 increase gel filtration column. The column was equilibrated with 1.2 column volumes of purification buffer (50 mM NaPi pH 7.4, 2 mM βME, 10% v/v glycerol, 0.05% DDM, 0.1 mM PMSF), and 1 ml fractions were collected. Eluted protein was spin-concentrated in 50 kDa cut off spin concentrators if necessary, flash frozen in liquid N_2_ and stored at −80 °C until use.

## Supplementary information

Supplementary Information.

## Data Availability

The data in this study are available from the corresponding author upon reasonable request.
