# Supplementary material for: Cell-free expression tools to study co-translational folding of alpha helical membrane transporters
Source: Sci Rep. 2020 Jun 4;10:9125. doi: 10.1038/s41598-020-66097-4 (PMC7272624; doi:10.1038/s41598-020-66097-4)
Supplement: Supplementary file 1 — Supplementary Information. [file 41598_2020_66097_MOESM1_ESM.docx]

**Cell-free expression tools to study co-translational folding of alpha helical membrane transporters**

Nicola J. Harris, Grant A. Pellowe and Paula J. Booth

^*^Corresponding author paula.booth@kcl.ac.uk

King’s College London, Department of Chemistry, Britannia House, 7 Trinity Street, London, SE1 1DB, UK

**Supplementary information**

Contents:

Supplementary Methods

Supplementary Figures

Supplementary Appendix Part 1- original gels from main figures

Supplementary Appendix Part 2- repeat experiments for protease digest gels

**Supplementary Methods**

**LacY and XylE reconstitution for protease digestion**

LacY and XylE were expressed using pET28a modified with a 10-His tag in *E. coli* BL21 AI cells, and purified as previously described [1] into 0.05 % dodecylmaltoside (DDM). Liposomes composed of 25:50:25 DOPC:DOPE:DOPG at 10 mg.ml^-1^ were made following the same procedure as that described for cell-free expression (see **Methods** in main paper). 1.2 % octylglucoside (OG) was added to the liposomes followed by incubation at room temperature for 20 min. 1 µg of LacY or XylE was added to the liposomes, and incubated for 1 hr at room temperature on a blood tube rotator. A detergent removal column (Pierce, ThermoFisher Scientific) was pre-incubated in 40 mM HEPES-KOH pH 7.6 prior to OG removal following the manufacturer’s instructions. 10 µl of the reconstitution was incubated with 20 ng thermolysin for 30 min, or with 10 ng thermolysin overnight, either with or without 2 % DDM. The samples were then analysed by SDS-PAGE, and an anti-His antibody used to detect protein. Results are shown in **Fig. S1**.

**Supplementary Figures**


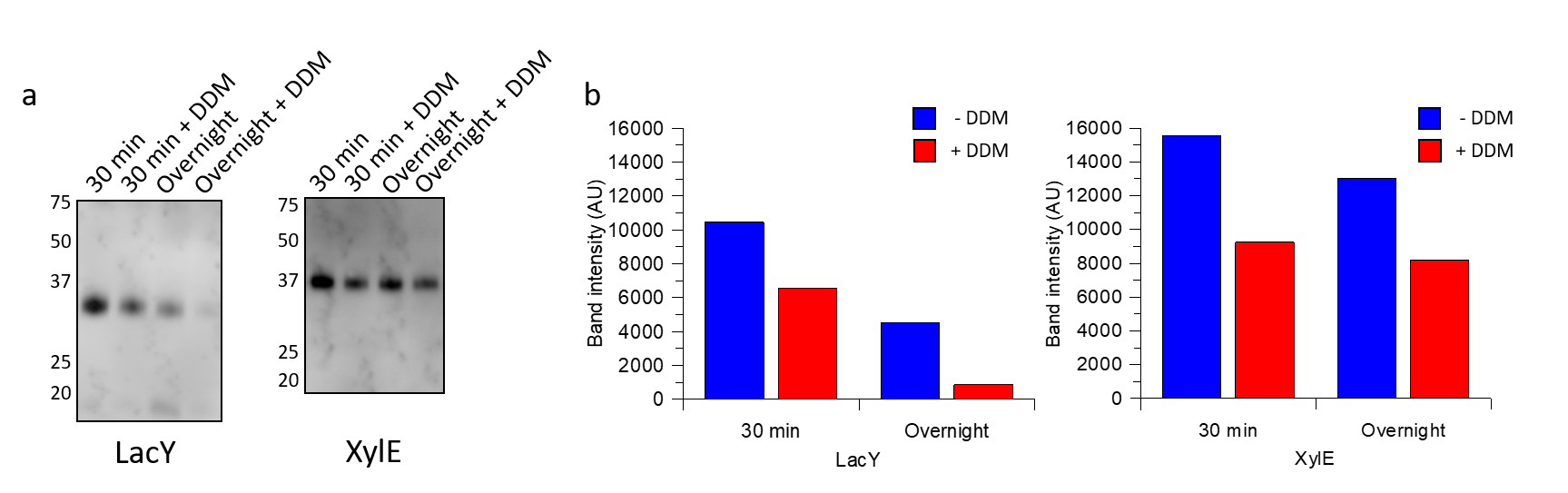


**Fig. S1 Thermolysin digest of LacY and XylE purified from *in vivo***

LacY and XylE were both expressed and purified from *E. coli*, and 1 µg of each was reconstituted into 25:50:25 DOPC:DOPE:DOPG liposomes (see **Supplementary Methods**). 10 µl of the reconstituted protein was incubated with 20 ng thermolysin for 30 min, or with 10 ng thermolysin overnight, either with or without 2 % DDM. The samples were then analysed by SDS-PAGE, and an anti-His antibody used to detect protein (**a**). The protein solubilised in DDM had fainter bands than those without DDM, indicating protection of protease sites when in liposomes. XylE was more resistant to proteolysis than LacY. The band intensities were quantified with ImageJ (**b**); bands in liposomes are in blue and with DDM in red. Representative gels are shown (see **Supplementary Appendix Part 2** for comparable gels from repeat experiments), gel intensities relate to the gel shown in the figure. The original uncropped gels are shown in the **Supplementary Appendix Part 1**, no other image adjustments have been made.


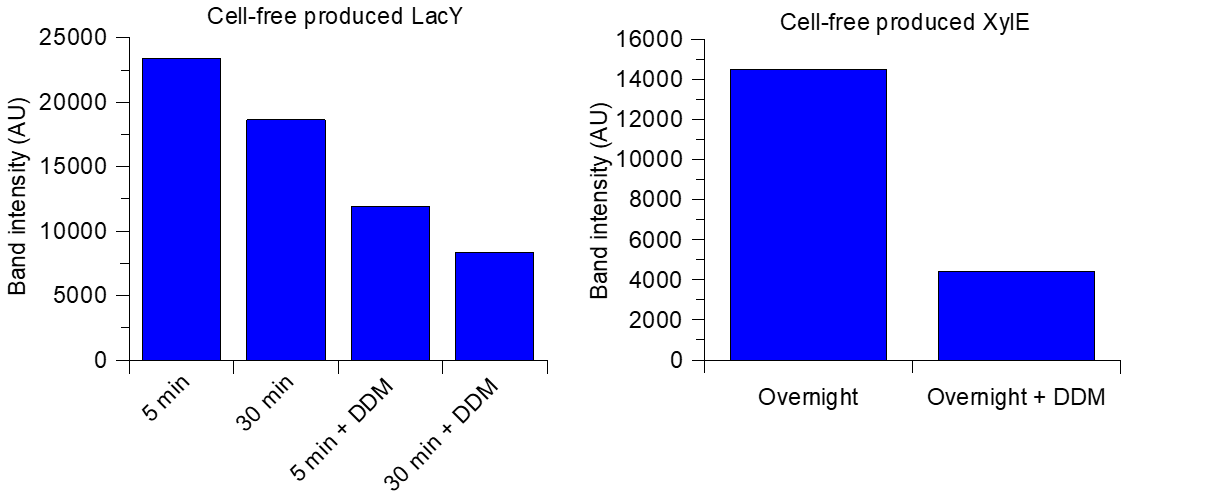


**Fig. S2 Band intensities from Fig 1E thermolysin digest**

LacY and XylE were made via cell-free expression in the presence of 25:50:25 DOPC:DOPE:DOPG liposomes. Following flotation on a sucrose gradient, LacY was incubated at 25 °C with 20 ng of thermolysin in 40 mM HEPES-KOH pH 7.6 for 5 or 30 min, either with or without 2 % DDM to solubilise the liposomes. XylE was incubated overnight at 4 °C with 10 ng thermolysin in 40 mM HEPES-KOH pH 7.6, either with or without 2 % DDM. Both transporters were more digested when solubilised in DDM, but protected from proteolysis when in liposomes, indicating stable insertion into the bilayer. Protein was detected via an anti-HA tag (LacY) or anti-His tag (XylE) antibody (**Fig. 1e**), and the bands were quantified in Image J (no image manipulations prior to analysis).


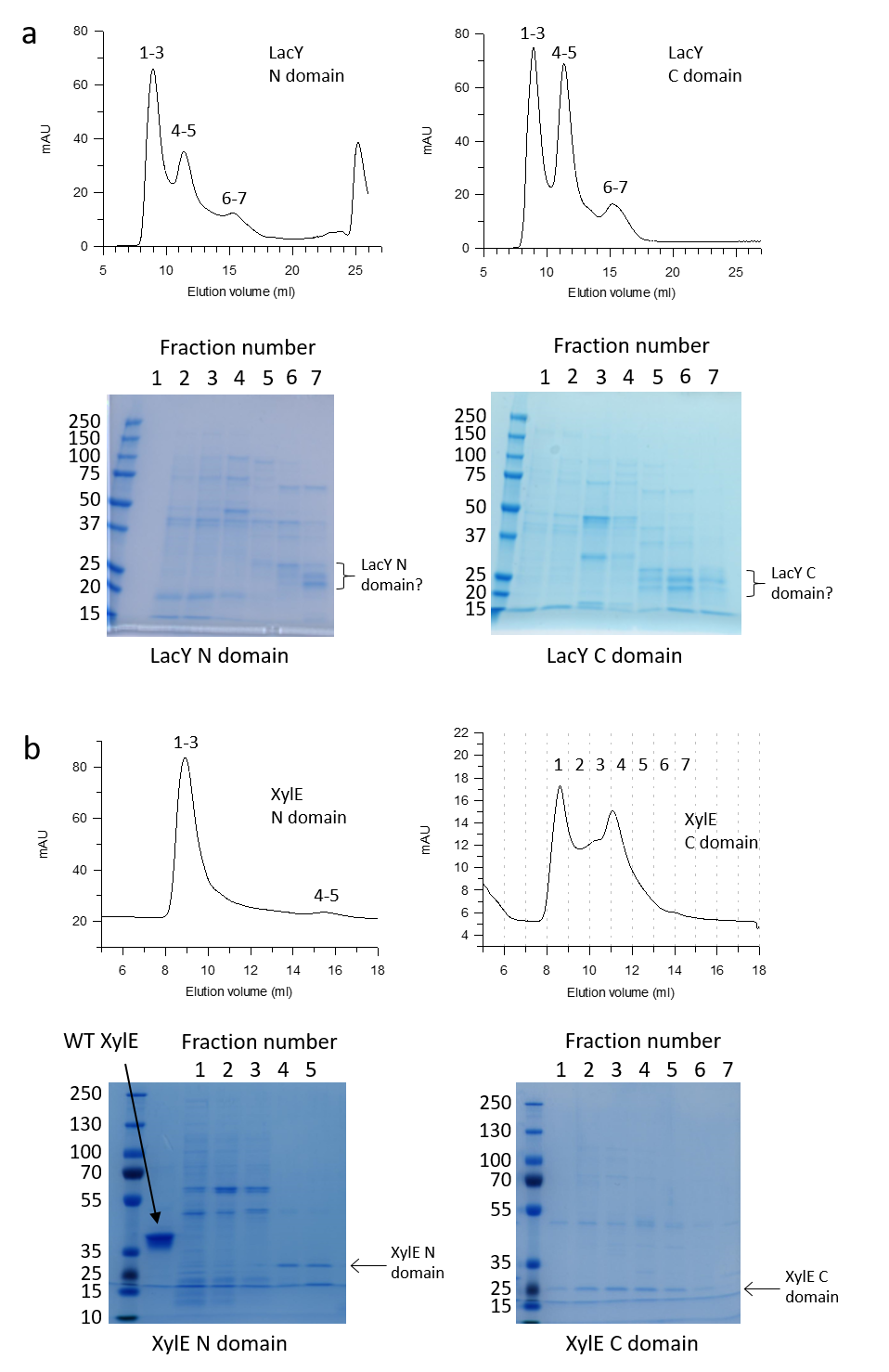


**Fig. S3 N and C domains cannot be purified from *in vivo***

The N and C domains of LacY (**a**) and XylE (**b**) were expressed separately and purified by His tag affinity chromatography followed by gel filtration. The Akta traces for the gel filtration column are shown with the corresponding SDS-PAGE gels for the fractions below. (**a**) LacY. Each domain should be in fractions 6-7, there is a large amount of degradation and higher order aggregates in these lanes. There are multiple bands present in these lanes, so it is unclear which corresponds to monomeric, folded protein. (**b**) XylE. The majority of the protein for each domain eluted in the void volume, therefore both were highly aggregated. Each domain should be in fractions 4-5 (N) or 6-7 (C), there is a large amount of degradation, some oligomerisation and a very low yield produced. WT XylE is also shown for comparison. The original gels are in this figure, cropped lanes are empty lanes and not shown for clarity.


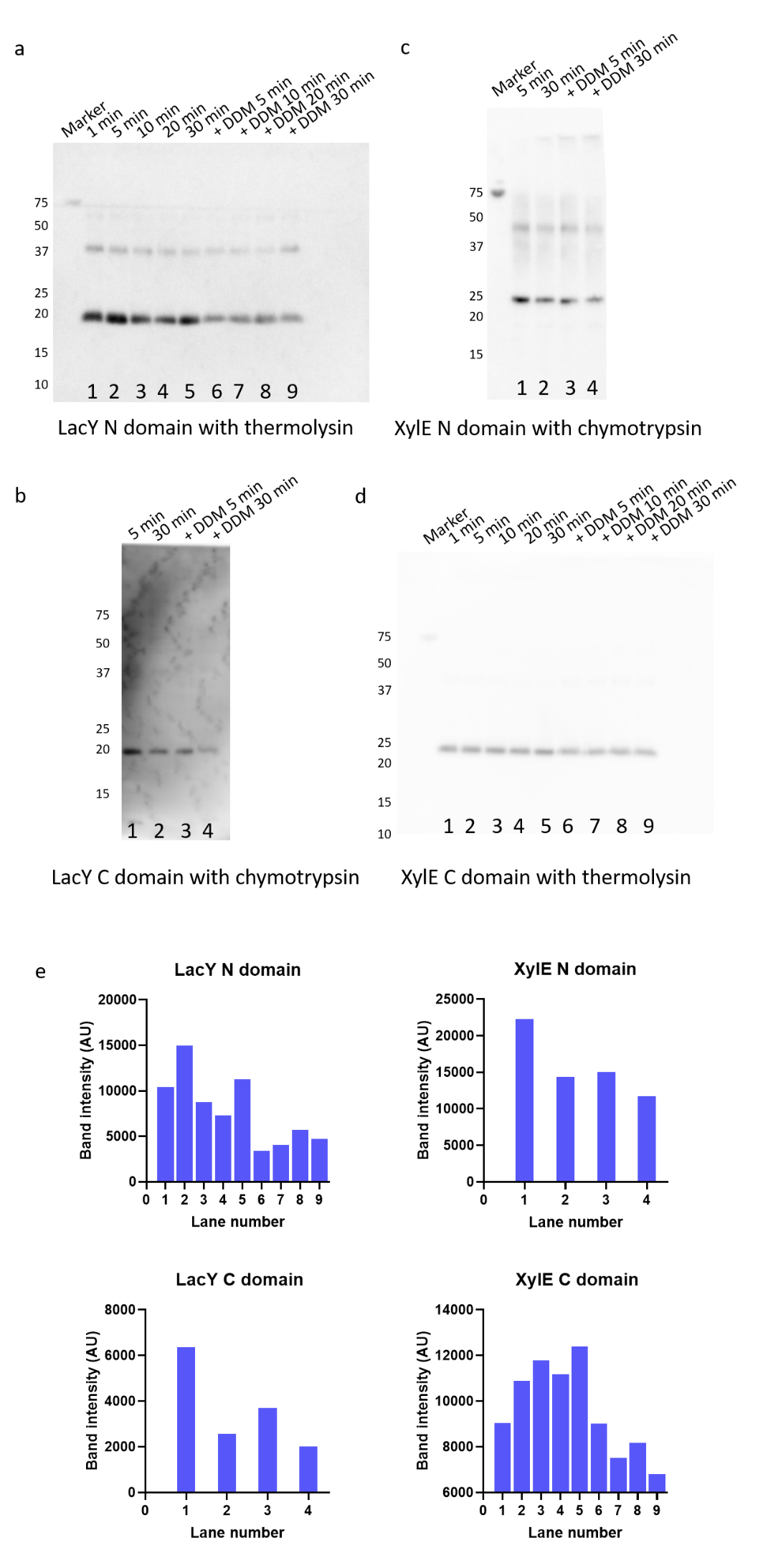


**Fig. S4 Original gels from Fig. 4**

Shown are the full range of time points from **Fig 4 a-d**. The graphs below (**e**) are the band intensities as quantified in Image J. At all of the time points measured, the corresponding time point with DDM has a lower band intensity. This demonstrates less protection of protease sites when solubilised in DDM, indicating incorporation into the liposome when DDM is absent. The lane numbers for each graph are the lanes left to right for each gel. Additional lanes in the gels shown in (**b**) and (**c**) have been cropped as they were an unrelated experiment, there are no other image adjustments. Representative gels are shown (see **Supplementary Appendix Part 2** for comparable gels from repeat experiments). The gel intensities in this figure correspond to these gels.


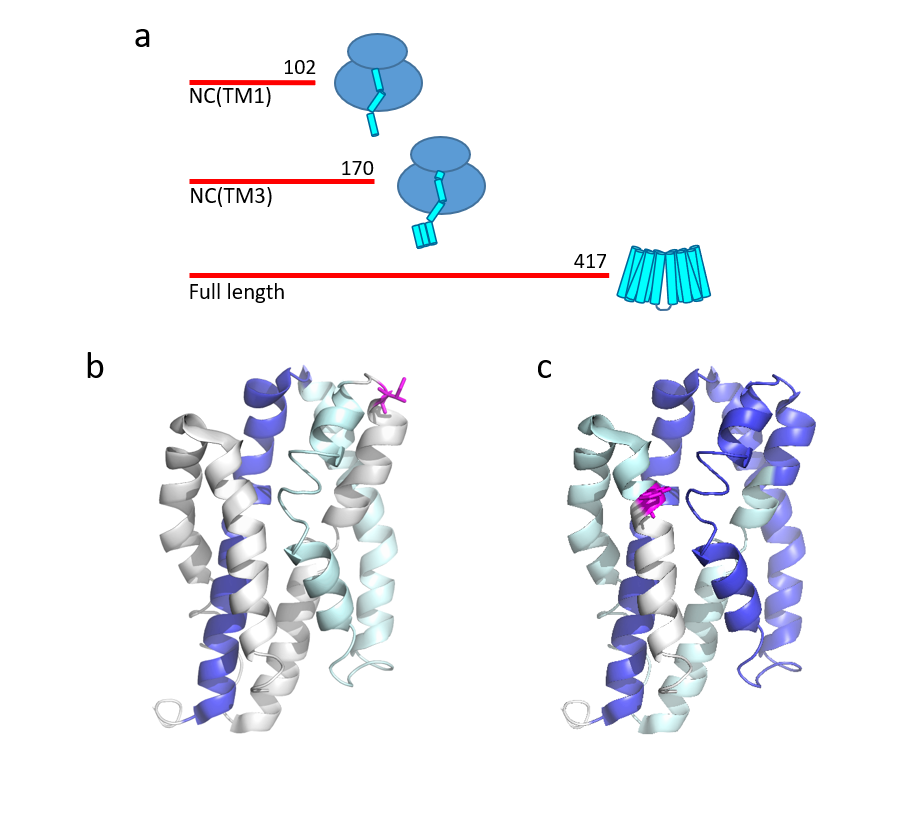


**Fig. S5 Sites chosen for pausing and restarting translation of LacY.** Sites for stopping and pausing the translation of LacY were made by inserting a single Trp codon at specific sites in the LacY sequence. There is room in the ribosome exit tunnel for ~60 amino acids if they are alpha helical [2], corresponding to approximately 2 TM helices. Sites were therefore chosen ~60 residues after one helix (NC(TM1), 103) and 3 helices (NC(TM3), 171) had emerged from the ribosome. An absence of Trp in the cell-free reaction therefore causes translation to stop at these sites and for the construct to remain ribosome-attached, and the resulting polypeptide can be analysed for association with the liposome. When ribosome-attached, the construct NC(TM1) has one helix outside the ribosome, and 2 helices inside. When it is ribosome-released, it has 3 TM helices. When ribosome-attached, the construct NC(TM3) has 3 helices and ~ 2 turns of helix 4 outside the ribosome, with the remainder of TM 4, TM 5 and ~ 2 turns of helix 6 inside the ribosome. When it is ribosome-released, it has 5 TM helices and ~ 2 turns of helix 6. Residue numbering is from the start codon. A schematic of the experimental design in shown in (**a**). (**b**) and (**c**) show the N domain of LacY with the locations of I103 (**b**) and W171 (**c**) highlighted in pink. The residues which exit the ribosome for each pause position are dark blue, those that remain in the ribosome are in light blue. Note that the position of W171 means that ~ 2 turns of TM4 will also have exited the ribosome.


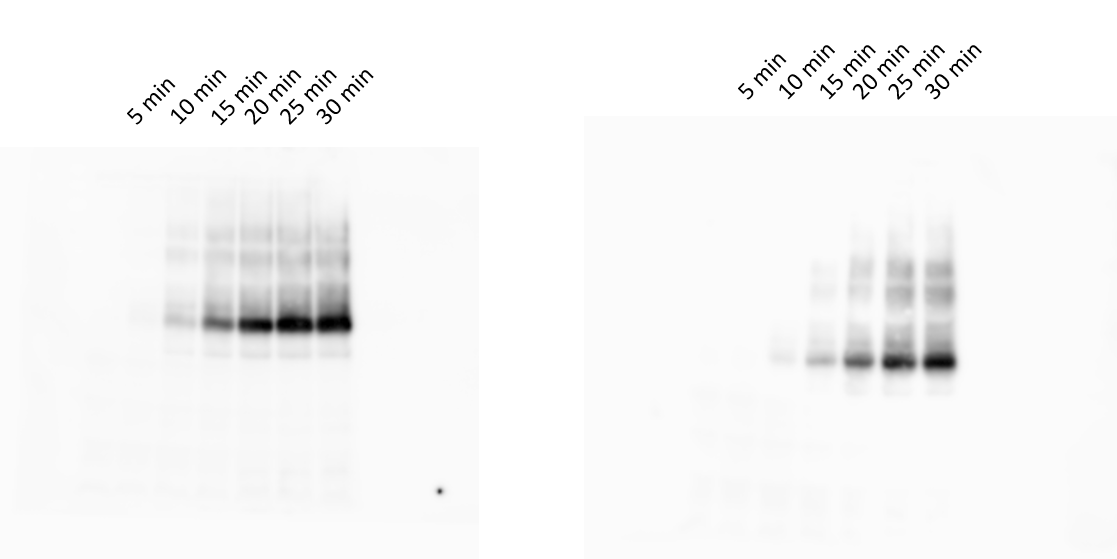


**Fig. S6 Rate of translation in PURExpress**

The constructs NC(TM1) (**left**) and NC(TM3) (**right**) were made in PURExpress at 30 °C in the presence of Trp in order to produce full-length protein. Samples were taken every 5 min. A band corresponding to full-length LacY appears for both constructs between 5 and 10 min.


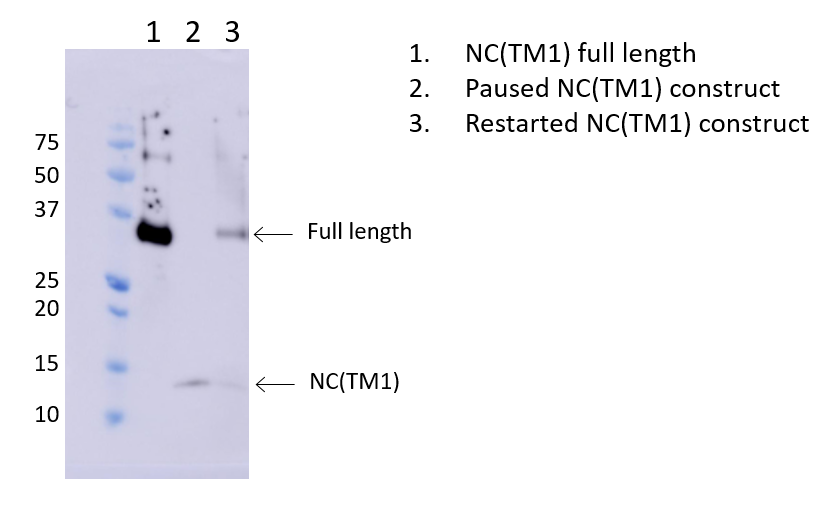


**Fig. S7 Reproducibility of pausing and restarting translation experiment**

Repeat of experiment in **Fig. 5a**. Lane 1 is the NC(TM1) construct when expressed with all amino acids in the cell-free reaction. Lanes 2 and 3 are the same as lane 1 and 2 in **Fig. 5a**. In this example, the same full length and paused bands appear, but the proportions are different and there is less oligomer. This demonstrates the reproducibility of the experiment, but also the difficulty transferring ribosome-attached protein during western blotting. Cropped lanes related to an unrelated experiment.


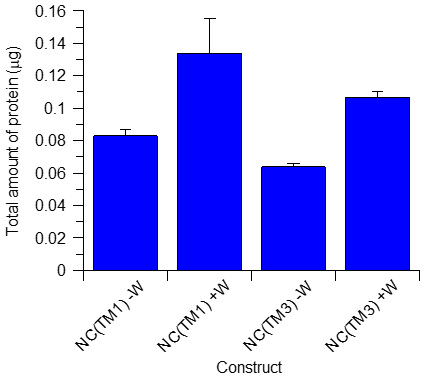


**Fig. S8 Expression of the NC(TM1) and NC(TM3) constructs**

Translation of the LacY NC(TM1) and NC(TM3) constructs was stopped by omitting Trp from the cell-free expression reaction (-W columns), or paused for 30 min before Trp was added back in (+W columns). The total amount of protein made in each cell-free expression reaction was quantified via LSC of incorporated [^35^S] Met. The amounts of protein measured do not relate to the band intensities observed by SDS-PAGE, aggregation is likely to be the cause.

**Supplementary Appendix Part 1- original gels from main figures**


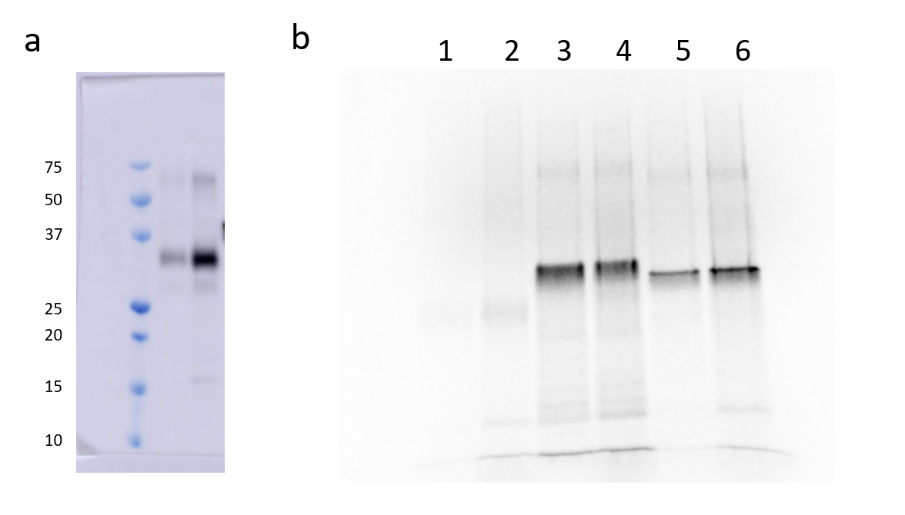


**Fig. S9 Original gels from Fig. 1d**

Shown are the uncropped images from **Fig. 1d**. (**a**) LacY western blot, bands were detected by an anti-HA tag antibody (additional lanes removed for clarity). The black and white version without markers is shown in **Fig. 1**, no other adjustments have been made. (**b**) XylE detected by phosphorimaging. Lanes 5 and 6 are those in Fig 1D. Lanes 1 and 2 are LacY top and bottom fractions of sucrose gradient, a western blot was chosen as the method of detection due to the low number of methionine residues in LacY. This gel also shows the LacY top fraction (lane 1) running as a very diffuse band. Lanes 3 and 4 another transporter and unrelated to the results in this manuscript.


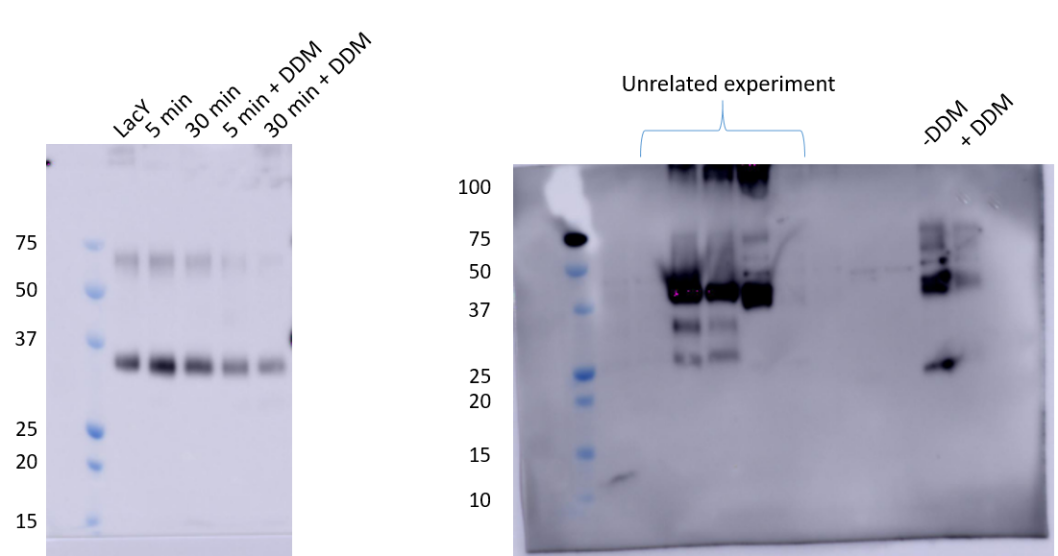


**Fig. S10 Original gels from Fig. 1e**

The left image is the original LacY protease digestion gel, the extra lanes related to a different experiment. The right image is the original gel from XylE protease digestion. The earlier lanes are an unrelated experiment and have been left in to include the size markers. No other image adjustments have been made.


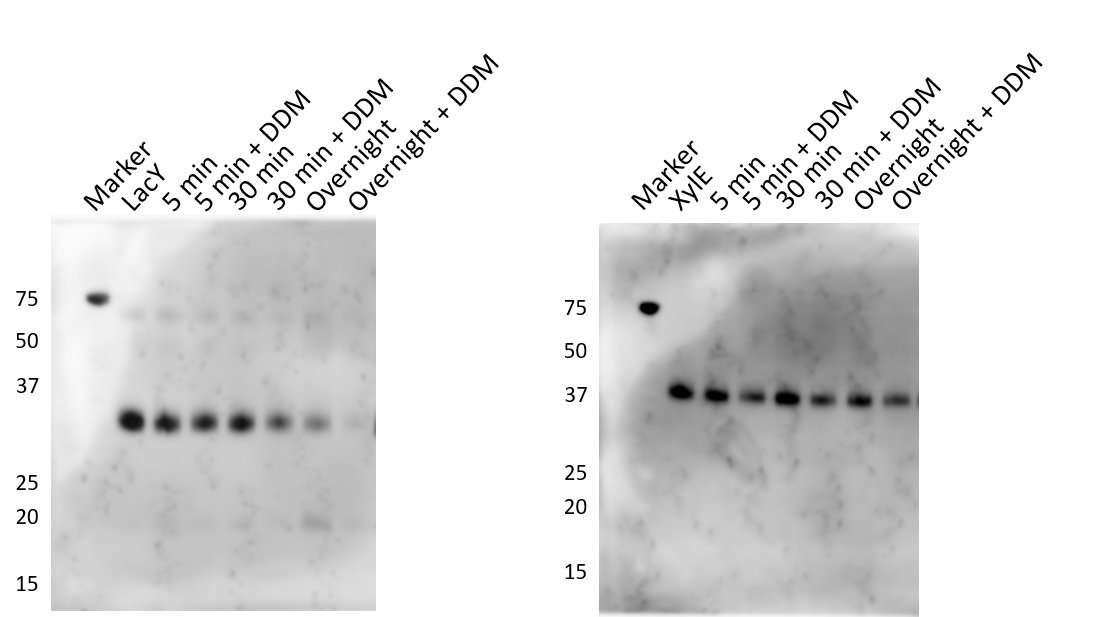


**Fig. S11 Original gels from Fig. S1**

These gels show extra timepoints for the gels shown in **Fig. S1**.


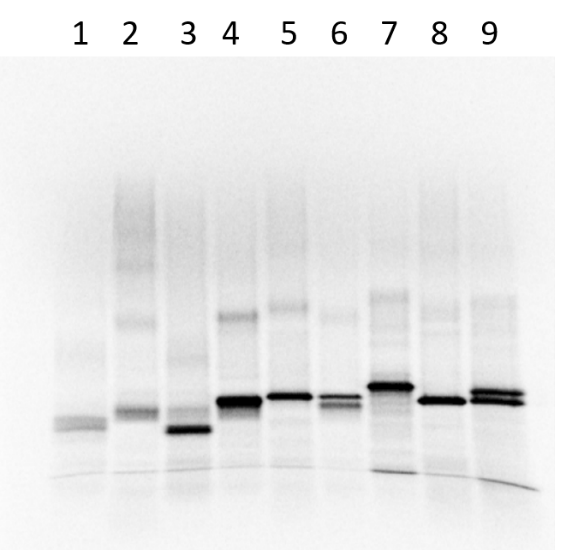


**Fig. S12 Original gel from Fig. 3**

The original, uncropped, image from **Fig. 3**. Lane numbering is the same as in the main figure, lanes 7-9 are from a different transporter not related to the results in this manuscript.

**Supplementary Appendix Part 2- repeat experiments for protease digest gels**


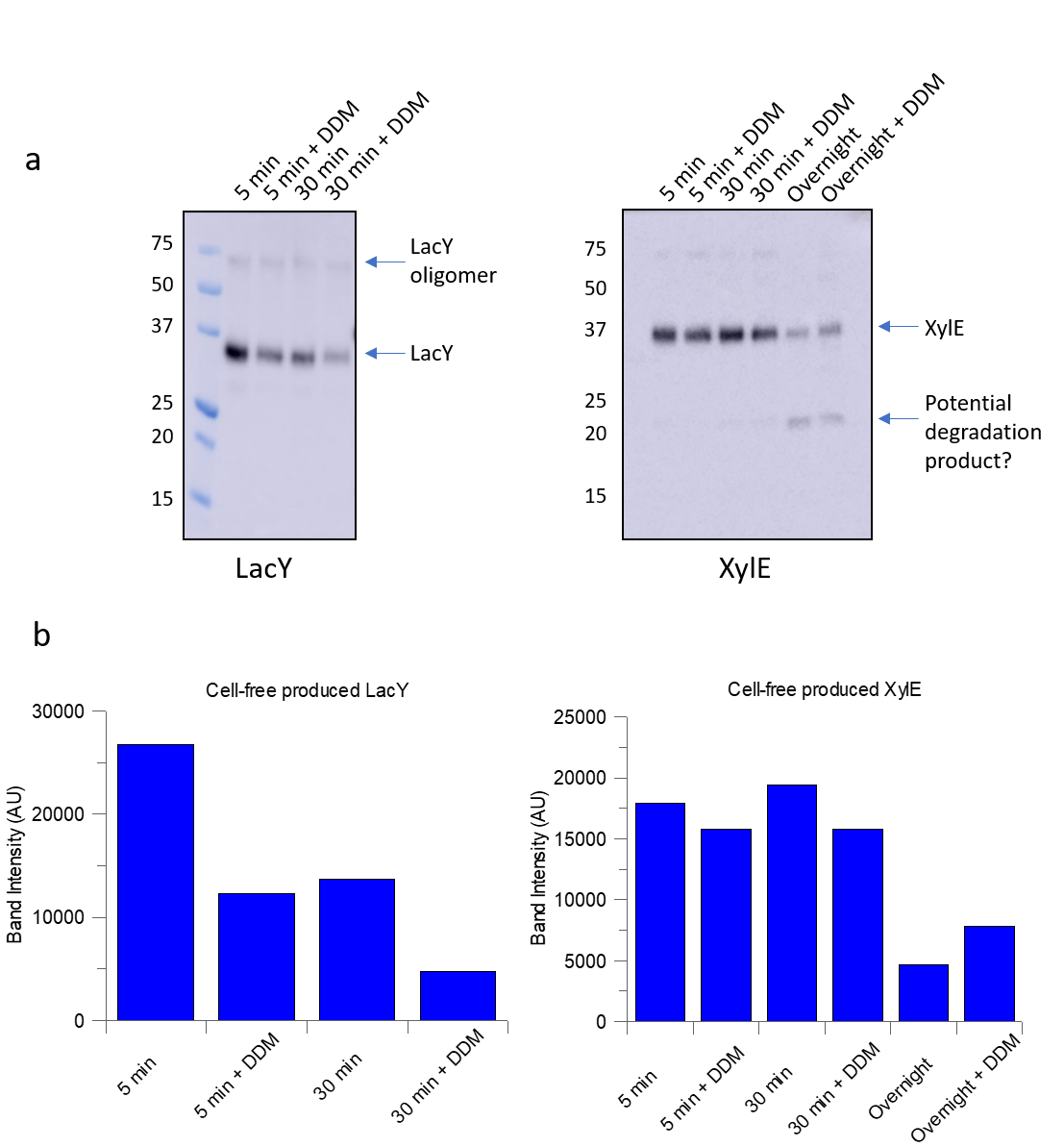


**Fig. S13 Repeat of experiment in Fig. 1e**

Shown in (**a**) are a repeat experiment of cell-free produced LacY and XylE when digested thermolysin at different timepoints. Cell-free produced protein solubilised in DDM is more digested than those in liposomes, indicating protection of protein in liposomes and the same trend as the gel shown in Fig. 1e. XylE has more timepoints than those shown in **Fig. 1e**. The band intensities in (**b**) relate to the gels in this figure.


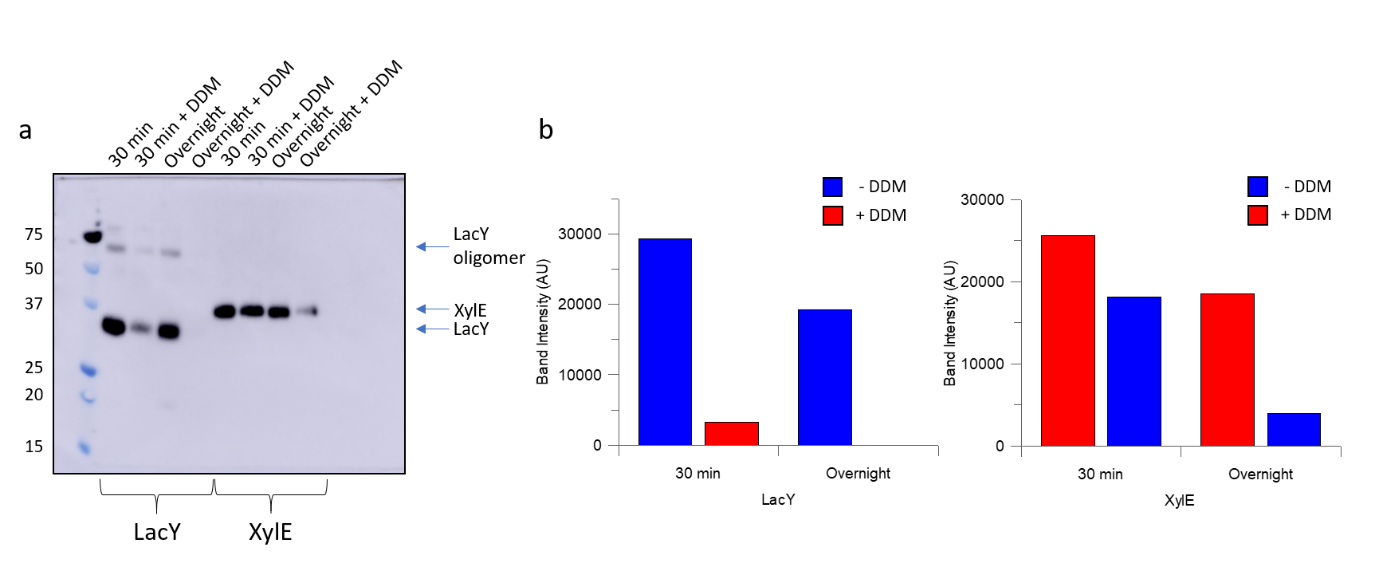


**Fig. S14 Repeat of experiment in Fig. S1**

This gel (**a**) shows reconstituted LacY and XylE digested with thermolysin, with and without DDM. The band intensities in (**b**) relate to the gel in this figure. This gel shows the same trend as that shown in Fig. S1. LacY has higher order oligomers (~60 kDa) which are also digested.


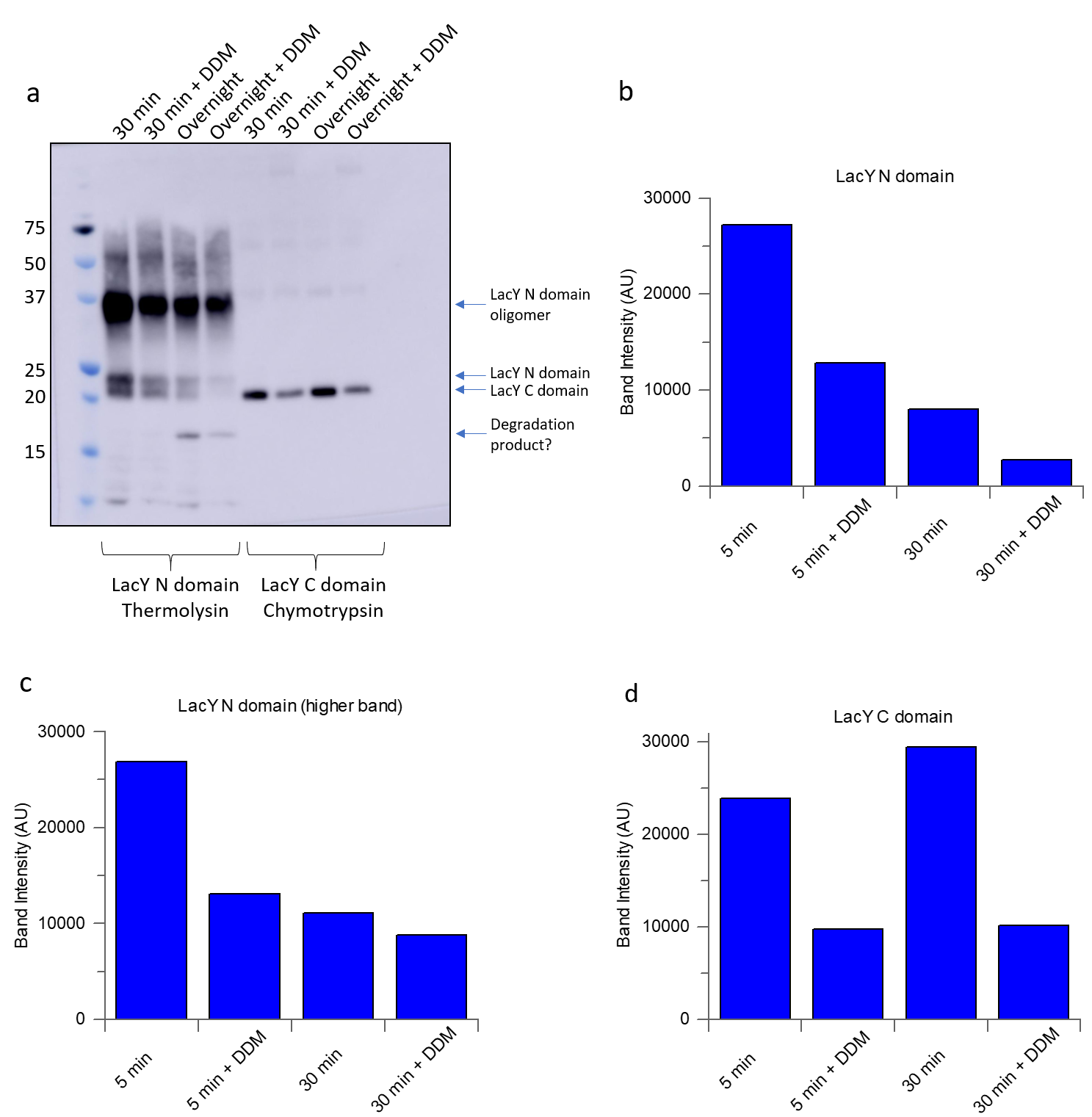


**
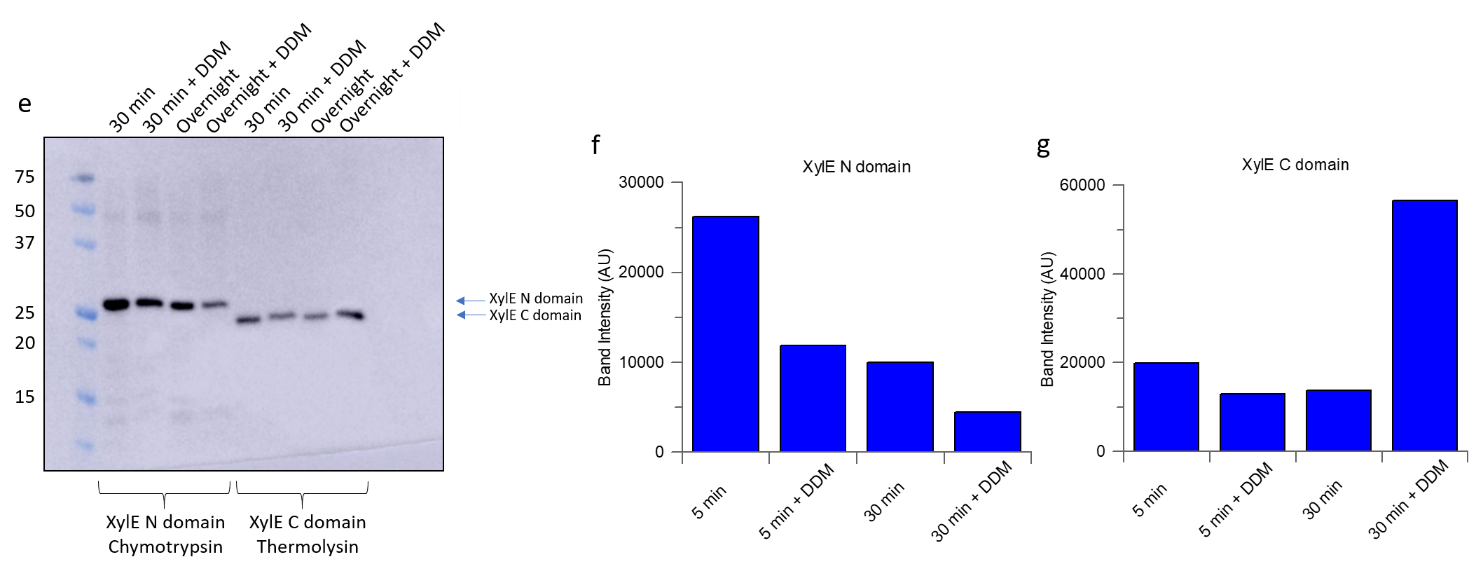
**

**Fig. S15 Repeat of experiment in Fig. 4 and Fig. S4**

The gel in (**a**) shows the LacY N domain digested by thermolysin, and the C domain digested by chymotrypsin for either 5 or 30 min with and without DDM. The corresponding gel intensities are in (**b-d**). In this example, the N domain has higher order oligomers (~ 37 kDa), which are also digested by protease (shown in **c**). The gel in (**e**) shows the XylE N domain digested by chymotrypsin and the C domain digested by thermolysin for either 5 or 30 min with and without DDM. The corresponding gel intensities are in (**f**) and (**g**). As in **Fig. 4** and **Fig. S4**, in all cases the protein solubilised in DDM is more digested than protein in liposomes.

1. Harris, N.J., et al., *Comparative stability of Major Facilitator Superfamily transport proteins.* Eur Biophys J, 2017. **46**(7): p. 655-663.

2. Bano-Polo, M., et al., *Transmembrane but not soluble helices fold inside the ribosome tunnel.* Nat Commun, 2018. **9**(1): p. 5246.
